# Shift of Dominant Species in Plant Community and Soil Chemical Properties Shape Soil Bacterial Community Characteristics and Putative Functions: A Case Study on Topographic Variation in a Mountain Pasture

**DOI:** 10.3390/microorganisms9050961

**Published:** 2021-04-29

**Authors:** Jinu Eo, Myung-Hyun Kim, Min-Kyeong Kim, Soon-Kun Choi

**Affiliations:** National Institute of Agricultural Sciences, RDA, Wanju 55365, Korea; wildflower72@korea.kr (M.-H.K.); kimmk72@korea.kr (M.-K.K.); soonkun@korea.kr (S.-K.C.)

**Keywords:** bacterial function, dominant species, nutrient, plant specific, topography

## Abstract

Reducing management intensity according to the topography of pastures can change the dominant plant species from sown forages to weeds. It is unclear how changes in species dominance in plant community drive spatial variation in soil bacterial community characteristics and functions in association with edaphic condition. Analysing separately the effects of both plant communities and soil chemical properties on bacterial community is crucial for understanding the biogeographic process at a small scale. In this paper, we investigated soil bacterial responses in five plant communities (two forage and three weed), where >65% of the coverage was by one or two species. The structure and composition of the bacterial communities in the different microbiome were analysed using sequencing and their characteristics were assessed using the Functional Annotation of Prokaryotic Taxa (FAPROTAX) and Kyoto Encyclopedia of Genes and Genomes (KEGG) pathways. Firmicutes and Planctomycetes responded only to one specific plant community, and each plant community harboured unique operational taxonomic units (OTUs) at the species level. There were a large percentage of uniquely absent OTUs for specific plant communities, suggesting that a negative effect is critical in the relationship between plants and bacteria. Bacterial diversity indices were influenced more by soil chemical properties than by plant communities. Some putative functions related to C and N recycling including nitrogen fixation were correlated with pH, electrical conductivity (EC) and nutrient levels, and this also implied that some biological functions, such as ureolysis and carbon metabolism, may decline when fertilisation intensity is reduced. Taken together, these results suggest that a shift of dominant species in plant community exerts individual effects on the bacterial community composition, which is different from the effect of soil chemical properties.

## 1. Introduction

Mountain pastures have been developed for agricultural purposes due to the intensification of lowland farming. However, these pasture habitats are subject to abandonment and decreased management due to their intensive labour requirements and for economic reasons [1]. Decreasing management intensity causes spatial variation in the soil chemical properties accompanied by vegetation succession [2]. Plant succession first involves early annual plants which are then replaced by perennial communities including shrubs [3,4]. Plant communities dominated by single or a few species may be formed due to changes in resource availability [5]. Pasture vegetation is mainly comprised of a few forage species and the substitution of dominant species greatly influences ecosystem processes at a local scale [6].

Soil bacteria are influenced by various biotic and abiotic factors. Plants represent a continuous supply of organic materials for soil communities shaping their structure over time [7,8]. In fact, through root exudates, they may create species-specific relationships favouring some species rather than others [9,10]. Moreover, plant residues may affect litter cycling and decomposition [11,12]. Conversely, soil bacteria can promote plant growth by changing the availability of nutrients and improve plant health by controlling pathogens [13,14].

The structure and composition of bacterial communities is highly heterogeneous and dependent on edaphic factors. The variability of soil chemical properties is critical for bacterial biodiversity as it can create various small-scale environments [15,16]. Soil pH, EC and nutrient levels have more profound effects than others [17,18]. Soil chemical properties also indirectly influence the soil ecosystem by inducing changes in plant community composition [19].

Plant communities have complex influences on soil bacteria in association with soil chemical properties. Results from previous studies are contrasting as to whether it is plant or soil characteristics that have a greater influence on soil bacteria [20,21]. Field researches based on surveys of natural communities have difficulties in dissociating the effect of plant from that of edaphic factors compared to experimental setups [22]. A different approach is needed to separate the individual effect of plant community other than correlational research, which is usually used for environmental factors. Analysing unique bacterial OTUs responded to specific plant is useful to understand the individual effect of the plant community [23].

Managing pastures from an ecological perspective is important for the conservation of resources and their environments. The role of species-rich grasslands, including semi-natural pastures, is increasingly recognised as important for the maintenance of biodiversity [24]. The interactions between above- and belowground environments drive soil ecosystem processes. In particular, the changes in the microbial community determined by soil properties during vegetation succession can shift soil ecosystem functions [25]. Hence, it would be useful to understand how the shift in dominant plant species influences soil bacterial functions for an ecological management of pastures.

This investigation aimed to determine how bacterial community characteristics and functions are influenced by plant community and how they are associated with soil chemical properties. Bacterial responses were compared in five plant communities having different dominant species. 16S rRNA sequencing was used to identify bacteria and to infer in silico their metabolic features. Uniquely responsive bacterial operational taxonomic units (OTUs) were analysed to discriminate the effect of plant community on bacteria from that of soil chemical properties.

## 2. Materials and Methods

### 2.1. Study Site

**Figure 1:**
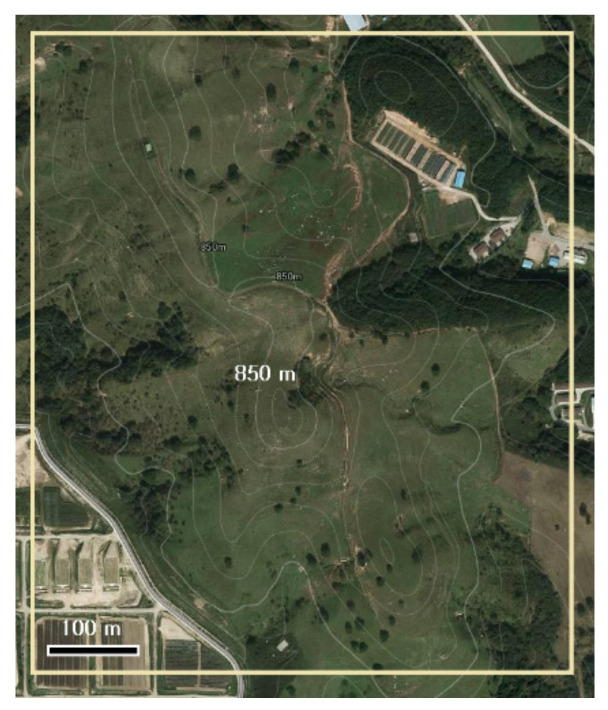
Topographic survey of study site. The photograph was obtained from Kakao Map (www.map.kakao.com, assessed on 21 April 2021).

The studied pasture was located in Pyungchang, Republic of Korea (37°41′04″ N, 128°44′02″ E). The sites average temperature (1981–2010) was 6.6 °C and the highest and lowest average monthly temperatures were 19.1 °C and −7.7 °C, in August and January, respectively. The annual precipitation was 1898.0 mm, and 1055 mm was concentrated between July and September. The altitude of the surveyed site was approximately 800–840 m, and the slope was from 4° to 45°. The characteristics of topography and plant communities are shown in Figure 1 and Table 1. In July 1956, 150 ha of pasture were reclaimed from forest and approximately 200 cows used it for feeding 4–5 times each year between May and October. Sowing was conducted with *Agrostis alba* (15 kg ha^−1^), *Phleum pratense* (3 kg ha^−1^), and *Poa pratensis* (2 kg ha^−1^). The soil texture was sandy loam comprised with 67.4% sand, 28.8% silt, and 3.8% clay. Synthetic fertiliser (21% N, 17% P and 21% K) was applied at 1.7 t ha^−1^ every year and no weeding was performed.

Vegetation composition and coverage was surveyed in the selected area (5.2 ha) according to the methods of Braun–Blanquet [26]. A total of five types of plant communities, two forage and three weed, were identified. Each plant community was named after one or two dominant species, which accounted for 65–100% of the cover. The sown forages were *A. alba* × *P. pratense* and *P. pratensis*. The natural herbs were *Festuca ovina* and *Rumex acetosella*, and the shrub association was *Spiraea miyabei* × *Spirae salicifolia*. Growth characteristics of plant species were assigned according to Lee [27] as shown in Table 1. Weed communities were formed where management was difficult according to topographic variation and showed various stages of vegetation succession. *F. ovina* is a tussock-forming perennial plant, which was mainly located in the highest areas of the site. This species originates from Europe, but also occurs naturally in South Korea. *R. acetosella* is distributed widely in Asia, Australia, Europe and North America, and is an early successional species that rapidly invades and colonises new sites [28]. *S. miyabei* and *S. salicifolia* was mainly found in the sloping areas of the site. The genus *Spiraea* is distributed in temperate and polar regions, and 14 species were observed in South Korea [29].

### 2.2. Soil Sampling and Analysis of the Chemical Properties

Soil sampling was conducted on 6 July 2015 when plant communities were fully formed. A total of three replicate plots (each measuring 5 × 6 m) were established for each of the selected five plant communities. The soil under stand of the plant assigned to dominant species was sampled at 0–10 cm depth to reflect the spatial effect of plant community because the sampled soils included dense roots [30]. Approximately 400 g of soil were collected with a soil sampler (5 cm in diameter) from 4 to 5 locations in each plot, and were homogenised to obtain a composite sample. The debris was carefully separated and the soil was passed through a 2-mm sieve. Then, 50 g of the soil was freeze-dried for 3 days and then preserved at −72 °C. The air-dried soil (30 g) was used to measure the soil chemical properties for each sample. Soil pH and EC were measured in a 1:5 (soil:water) suspension using a pH meter and conductivity meter, respectively. NO_3_^−^ and NH_4_^+^ were extracted with 2 M KCl, and the available P_2_O_5_ was measured using an acetate–lactate buffer. The concentrations of these nutrients were determined using a SmartChem autoanalyser (Westco, Italy) according to the manufacturer’s instructions. The total C and N levels were determined using a CN analyser (Vario Max CN, Elementar, Hanau, Germany), and the C/N ratio was calculated from the total C and total N. These analyses were conducted in duplicate.

### 2.3. DNA Extraction and PCR

Genomic DNA was extracted according to Chun et al. [31]. Freeze-dried soil (0.5 g) was extracted using a FastDNA SPIN Kit for Soil (MP Biomedicals, Solon, OH, USA). DNA was amplified using a PharmaTech and GeneAmp PCR System 9700 (Applied Biosystems, Foster City, CA, USA). The hypervariable V1-3 region of the bacterial 16S rRNA gene was amplified using V1-9F (5′-CCTATCCCCTGTGTGCCTTGGCAGTC-TCAG-AC-AGTTTGAT CMTGGCTCAG-3′) and V3-541R (5′-CCATCTCATCCCTGCGTGTCTCCGAC-TCAG- AGAGCTG-AC-WTTACCGCGGCTGCTGG-3′) [31]. DNA was not extracted in replicates and the concentration of the extracted DNA was more than 20 ng μL^−^^1^. Each 50-μL PCR mixture was composed of 1-μL (1:10 dilution) isolated DNA, 5-μL of PCR buffer (1×), 1 μL of each deoxyribonucleoside triphosphate (100 mM), 2 μL of forward and reverse primers (20 pM), and 0.25-μL Taq polymerase (5 U μL^−^^1^). The PCR products were purified using a QIAquick PCR Purification Kit (QIAGEN, Hilden, Germany). The amplification protocol consisted of an initial denaturation step for 5 min at 94 °C, followed by 30 cycles of 30 s at 94 °C, 45 s at 55 °C, and 90 s at 72 °C.

### 2.4. Pyrosequencing

Pyrosequencing was performed according to Chun et al. [31]. The sequences were determined using a 454 GS FLX Titanium Sequencing system (Roche, Branford, CT, USA). For pyrosequencing, 0.5 μg of the PCR product was used. Sequencing reads that were shorter than 300 bp in length or contained two or more unresolved nucleotides were removed. Average read length was 419.1 bp and the highest was 521 bp. The AmpliconNoise pipeline was used to correct pyrosequencing errors. OTU clustering was based on the CD-HIT algorithm with a 97% cut-off, and classification was conducted using the EzBioCloud (www.ezbiocloud.net; accessed on 1 April 2021). The mean numbers of valid reads and OTUs per plot were 6656.5 and 1951.3, respectively (Table 2). Taxonomic classifications were determined using a criterion of 94% identity for genus and 75% identity for phylum. To assess the plant-specific effect on bacterial major OTUs (>0.01%), uniquely responsive OTUs were counted. Uniquely present and absent OTUs were those that were found or missing for only one type of plant community, respectively.

### 2.5. Expectation of Bacterial Function

For taxonomic expectation of function, the bacterial communities were analysed using the FAPROTAX database, and their functions were estimated by summing the relative abundances assigned to each function [32]. For genetic expectation of function, the 16S rRNA sequencing reads were calculated using the EzBioCloud MTP pipeline. The pipeline predicts Kyoto Encyclopedia of Genes and Genomes(KEGG)-based pathways using the same algorithm as PICRUSt [33].

### 2.6. Data Analysis and Statistics

Pyrosequencing data were analysed with a cut-off value of 97% sequence similarity to assign phylotypes. The numbers of uniquely present or absent OTUs in three replicate plots were summed to calculate the percentage of uniquely responsive OTUs in a major group (>0.01%). One-way ANOVA followed by Tukey’s test (*p* < 0.05) was conducted to detect significant differences among the bacterial community compositions and soil chemical properties. Each sample was not sequenced in replicates in relation to sequencing results. Spearman’s rank correlation coefficients were calculated to test correlations between the soil chemical properties and the OTUs assigned to genera or species. These tests were performed using SAS v9.1 (SAS Institute Inc., Cary, NC, USA). Canonical correspondence analysis (CCA) was conducted to further analyse the relationships between chemical properties with the dominant bacterial phyla. Analysis of similarities (ANOSIM) was performed to determine the significance of the differences between bacterial communities using the vegan package of R (version 3.4.3) [34]. Shannon and Chao1 indices were calculated using EzBioCloud to estimate the alpha diversity. Beta diversity (the distance between bacterial communities) was analysed using the Fast Unifrac online tool for principal coordinate analysis (PCoA) at the OTU level.

## 3. Results

### 3.1. Soil Chemical Properties and Plant Communities

The plant communities differed greatly in terms of their soil chemical properties (Table 2). However, there was no obvious difference between the seeded and native communities. The EC was significantly higher in *P. pratensis* than in *S. miyabei* × *S. salicifolia*, and the concentrations of NO_3_^−^ and available P tended to be lower in *S. miyabei* × *S. salicifolia*.

### 3.2. Pyrosequencing Analysis of the Bacterial Communities

**Figure 2:**
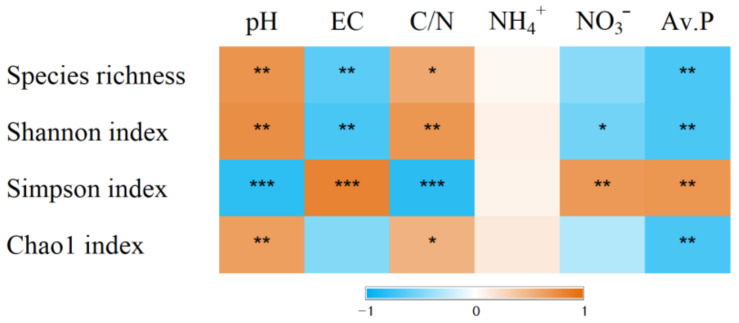
Spearman’s correlation coefficient between diversity indices and soil chemical properties (*n* = 15). The colour indicates the value of correlation coefficient (−1 < *r* < 1). *, **, and *** indicate significant correlation at *p* < 0.05, *p* < 0.01, *p* < 0.001.

**Figure 3:**
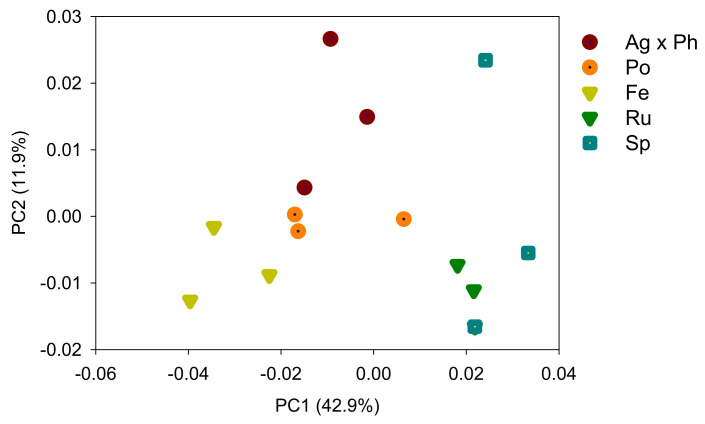
Microbial community analysed by principal coordinate analysis of Unifrac distances. Ag × Ph, *A. alba* × *P. pratense*; Po, *P. pratensis*; Fe, *F. ovina*; Ru, *R. acetosella*; Sm × Ss, *S. miyabei* × *S. salicifolia*.

The percentage of uniquely absent OTUs was 3.3% for dominant species (>0.1%) and 30.0% for subdominant species (>0.01%). In contrast, the percentage of uniquely present OTUs was zero for the dominant species and 1.8% for the subdominant species (Table 3). The alpha diversity indices were influenced by the plant communities (Table 3). The numbers of OTUs and Shannon indices were low for *P. pratensis* and *S. miyabei* × *S. salicifolia*. The species richness and Shannon index were positively correlated with pH and the C/N ratios, and negatively correlated with EC and available P (Figure 2). PCoA was used to explore the plant-specific effects, and the bacterial communities in the five plant regimes were separated by PC1 and PC2 (Figure 3). ANOSIM revealed that the bacterial communities differed significantly depending on plant communities (R = 0.847, *p* = 0.0001).

### 3.3. Responses of Bacteria at the Phylum, Genus and Species Levels

**Figure 4:**
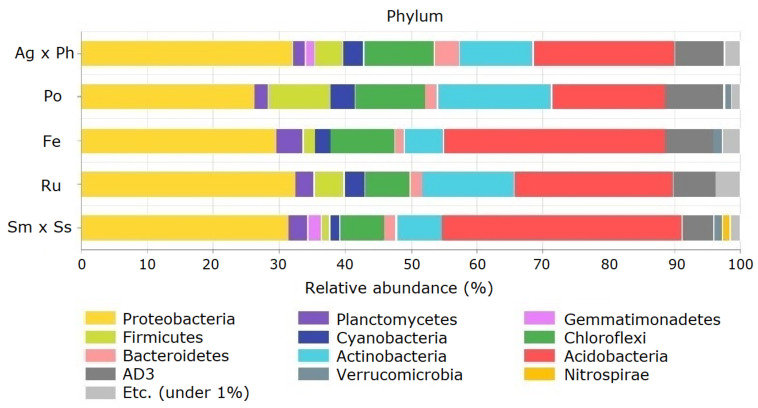
Relative abundance of soil bacteria at the phylum level in the pasture. Ag × Ph, *A. alba* × *P. pratense*; Po, *P. pratensis*; Fe, *F. ovina*; Ru, *R. acetosella*; Sm × Ss, *S. miyabei* × *S. salicifolia*.

**Figure 5:**
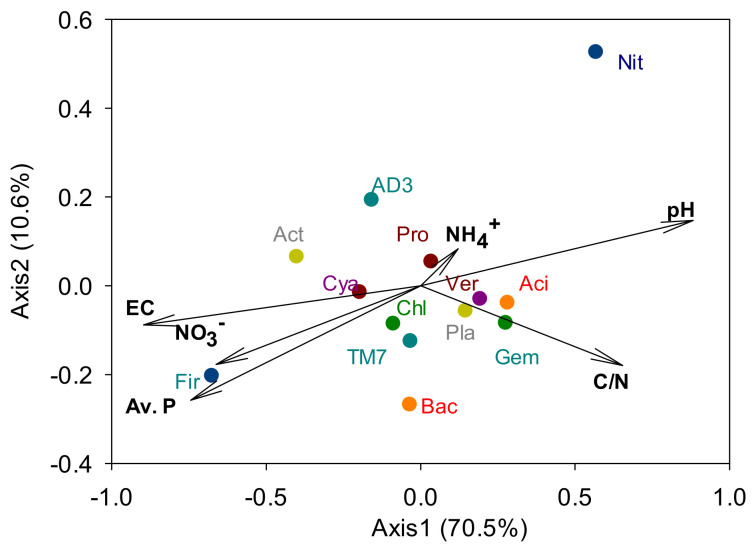
Canonical correspondence analysis between soil chemical properties and dominant bacterial phyla. Aci, Acidobacteria; Act, Actinobacteria; AD3, a candidate division; Bac, Bacteroidetes; Chl, Chloroflexi; Cya, Cyanobacteria; Fir, Firmicutes; Gem, Gemmatimonadetes; Nit, Nitrospirae; Pla, Planctomycetes; Pro, Proteobacteria; TM7, a candidate division; Ver, Verrucomicrobia.

Proteobacteria, Acidobacteria, Actinobacteria, Chloroflexi and AD3 were the dominant phyla as they made up >5% of each community on average, and accounted for 82.4% of the total abundance (Figure 4). Some phyla responded only at one specific plant community. The relative abundance of Firmicutes was highest with *P. pratensis*, and Planctomycetes levels were highest with *F. ovina*. Among the minor phyla, OD1 were absent only from *P. pratensis*, and Deinococcus–Thermus were present only with *A. alba* × *P. pratense* and showed an average relative abundance of 0.02%. Meanwhile, relative abundance of Cyanobacteria was lower with *S. miyabei* × *S. salicifolia* than with *P. pratensis*. CCA showed that some dominant phyla were strongly associated with soil chemical properties (Figure 5).

**Figure 6:**
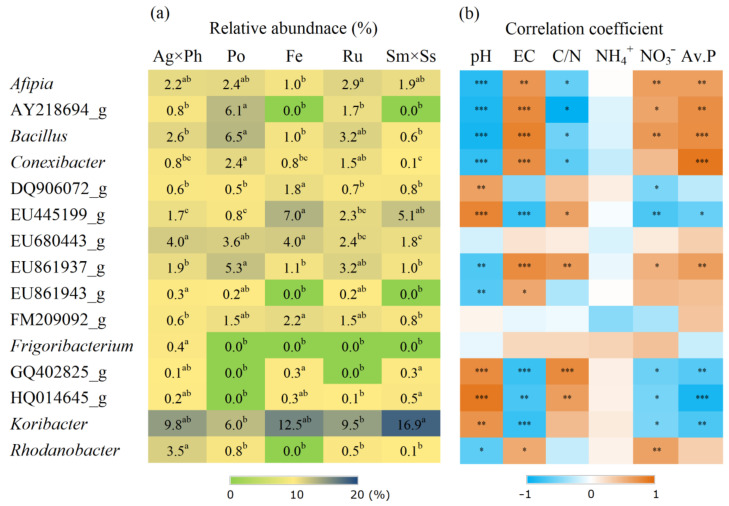
Relative abundance of 15 major bacterial genera showed significant difference and its correlation with soil chemical properties. (**a**) Relative abundance of bacteria; (**b**) Spearman’s correlation coefficient. Ag × Ph, *A. alba* × *P. pratense*; Po, *P. pratensis*; Fe, *F. ovina*; Ru, *R. acetosella*; Sm × Ss, *S. miyabei* × *S. salicifolia*. Different letters in the same row indicate significant differences according to Tukey test (*n* = 3, *p* < 0.05). *, **, and *** indicate significant correlation at *p* < 0.05, *p* < 0.01 and *p* < 0.001, respectively.

Diverse responses were found at the genus level, and some groups exhibited responses that were highly plant-specific (Figure 6). For example, the relative abundances of DQ906072_g and AY218694_g were highest with *F. ovina* and *P. pratensis*, respectively. Moreover, levels of *Rhodanobacter* were highest with *S. miyabei* × *S. salicifolia*. There were two genera comprising HQ014645_g (Proteobacteria) and GQ402825_g (Planctomycetes) who were absent only from *P. pratensis*, while EU861943_g (Gemmatimonadetes) was absent from *F. ovina* and *S. miyabei* × *S. salicifolia*. In contrast, *Parafrigoribacterium* was only present with *A. alba* × *P. pratense*. Meanwhile, the Spearman’s correlation coefficients showed that many bacterial groups had significant correlations with the pH, EC and nutrient levels (Figure 6).

The relative abundance of some species only increased with one specific plant community, and this phenomenon was observed with all five plant communities (Table 4). A total of two OTUs, FJ536874_s and AJ544784_s, comprised up to 10.3% of the relative abundance bacteria in *P. pratensis*, while FJ466008_s and *Sporosarcina soli* increased only with *P. pratensis*, and *Parafrigoribacterium mesophilum* was found only with *A. alba* × *P. pratense*.

### 3.4. Putative Functions of Soil Bacteria

**Figure 7:**
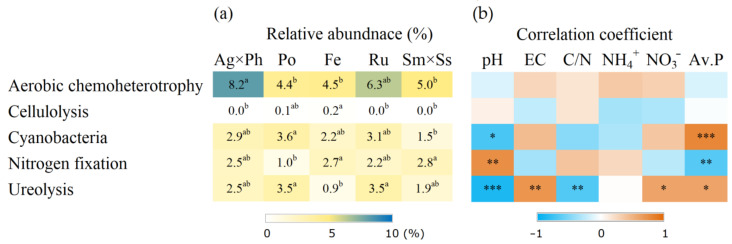
Putative functions of soil bacteria showed significant difference according to FAPROTAX and their correlation with soil chemical properties. (**a**) Relative abundance of bacteria; (**b**) Spearman’s correlation coefficient. Ag × Ph, *A. alba* × *P. pratense*; Po, *P. pratensis*; Fe, *F. ovina*; Ru, *R. acetosella*; Sm × Ss, *S. miyabei* × *S. salicifolia*. Different letters in the same row indicate significant differences according to Tukey test *(n* = 3, *p* < 0.05). *, **, and *** indicate significant correlation at *p* < 0.05, *p* < 0.01 and *p* < 0.001, respectively.

**Figure 8:**
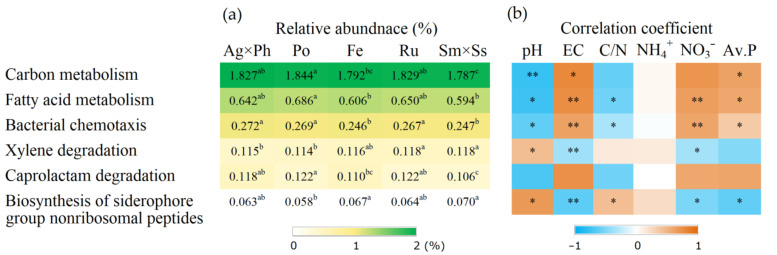
Putative functions of soil bacteria showed significant difference according to KEGG pathway and their correlation with soil chemical properties. (**a**) Relative abundance of bacteria; (**b**) Spearman’s correlation coefficient. Ag × Ph, *A. alba* × *P. pratense*; Po, *P. pratensis*; Fe, *F. ovina*; Ru, *R. acetosella*; Sm × Ss, *S. miyabei* × *S. salicifolia*. Different letters in the same row indicate significant differences according to Tukey test *(n* = 3, *p* < 0.05). * and ** indicate significant correlation at *p* < 0.05 and *p* < 0.01, respectively.

Putative function analysis using FAPROTX showed that there were five distinct functions that significantly differed in the plant communities (Figure 7). N metabolism, including nitrogen fixation and ureolysis, was correlated with pH and available P. Putative function analysis based on KEGG pathways showed that six functions were significantly influenced by plant communities (Figure 8). Some of these functions were significantly correlated with soil chemical properties, including pH, EC and nutrients.

## 4. Discussion

### 4.1. Vegetation and Environment

The dynamics of vegetation change appear to be related to the soil properties and topography. *F. ovina* has a slow growth rate and tolerates dry and nutrient-poor soils, which explain its dominant colonisation of the ridge area [35]. Moreover, the species is restricted by N fertilisation in early succession stage and more competitive in unfertilised condition [36]. *R. acetosella* is known to be a mid-successional plant, and was associated with acidic soils, and this is in agreement with the results of this study [37]. *S. miyabei* and *S. salicifolia* were observed mainly on the slopes, and the emergence of these shrubs was partly attributed to reduced feeding and management.

### 4.2. Bacterial Groups Responed Only to Speicific Plant Community

At the phylum level, Firmicutes and Planctomycetes were significantly influenced only by *P. pratensis* and *F. ovina*, respectively, which indicated clear associations between certain plants and bacteria. Relative abundance of Firmicutes was the highest with *P. pratensis*. It is in accordance with a previous work which reported that Firmicutes increased by the planting of *P. pratensis* in a pot experiment [38]. Moreover, relative abundance of Firmicutes was higher with monoculture of *P. pratensis* than with the mixture of *P. pratensis* and *Festuca rubra* [39]. This suggested that dominance of *P. pratensis* in plant community might be favourable for Firmicutes. Relative abundance of Planctomycetes was the highest with *F. ovina* but little is known about the direct associations between these two species.

Enrichment of Firmicutes and Planctomycetes with *P. pratensis* and *F. ovina* are partly explained by the soil nutrient condition in each plant community because two phyla are assigned to the oligotrophic and copiotropic group, respectively [40]. However, these factors could not fully explain why their levels were highest only with one specific plant community in this investigation, as there was no gradient in the variation in the values of these OTUs between different plant communities in response to soil chemical properties. Thus, it was shown that plant specific responses occur between two organisms, irrespective of the soil chemistry. In addition, it is notable that particularly two phyla were negatively correlated with root biomass in a previous study [22]. Their enrichment in different plant communities indicated that they were more influenced by plant factors other than root biomass. It can be explained by previous works that plants form relationships with specific bacteria and create a distinct bacterial community via root exudates [41,42].

At the species level, some OTUs enriched only with a specific plant community, and the phenomenon was commonly observed with the five plant communities. This implied that changes in dominant plant species might harbour specific soil bacteria. Meanwhile, an average of 5.2% of the dominant OTUs in five plant regimes were uniquely absent from specific plant communities. This indicates that much of the plant-specific effects might be caused by the suppression of bacteria rather than the promotion of specific bacteria. Plants produce suppressive compounds that have different effects on soil microbes, and the diversity of bacteria is more sensitive to changes in these materials than that of fungi [43]. A negative effect of plant on microorganisms may indirectly affect competitions between plants [44]. Plant communities seemed to shape bacterial community composition via both promoting and suppressing effects.

### 4.3. Bacterial Functional Groups Influenced by Plant Communities

Bacterial groups that responded only to one specific plant community are involved in various biological functions in relation to plant growth and nutrients. Planctomycetes can induce resistance to pests and degrade plant-derived polymers [45,46]. *Rhodanobacter* is involved in the denitrifying process that leads to N losses, and the dominance of *Rhodanobacter* with the specific plant species can be explained by the utilisation of the root exudates [47]. AJ544784_s belongs to the genus *Bacillus*, and some species of this genus promote the growth and yield of *P. pratensis* [48]. Moreover, *P. pratensis* promotes its own nutrient supply by stimulating the soil microbial population [49].

Differences in bacterial functions were expected based on the comparisons of the two plant communities. Relative abundance of cyanobacteria was higher with *P. pratensis* than that with *S. miyabei* × *S. salicifolia*. This is consistent with the results of a previous study showing that cyanobacteria were predominant around grasses but rare around shrubs [50]. Cyanobacteria are involved with nitrogen fixation via their symbiotic relationships with plants, wherein they promote plant growth by supplying nitrogen as well as improving resistance to environmental stress [51,52,53]. In addition, relative abundance of *Bacillus* was 6.8 times higher with *P. pratensis* than with *F. ovina*. It is well known that the genus *Bacillus* has the potential to promote plant growth and inhibit pathogens [8,54]. White et al. [55] reported that *Bacillus amyloliquefaciens* improved the utilisation of N when found in symbiosis in the rhizosphere of *Poa annua,* the same genus with *P. pratensis*.

### 4.4. Effects of Soil Chemical Properties on the Bacterial Community Composition at the Genus Level

Bacteria exhibited various responses to soil chemical properties at the genus level. *Koribacter* is difficult to culture, and it grows slowly on complex and low nutrient media, and may have diverse response to environmental changes [56]. *Koribacter* belongs to Acidobacteria, and the phylum is known to flourish at low pH [57]. Their negative correlation with nutrients can be explained by their preference for oligotrophic environments [41]. Moreover, this genus was negatively affected by available phosphorus in a previous report where the genus was enriched by repeated N and K fertilisation without P [17]. These results emphasise the importance of P availability in the ecology of *Koribacter*. In contrast, *Afipia*, *Bacillus* and *Conexibacter* are assigned to the copiotrophic group that grows in nutrient rich soil, and their positive correlation with nutrients reflects ecological niche [40].

### 4.5. Diversity of the Bacterial Communities

Changes in dominant plant species affect the availability of resources, and each species contributes to the function of the microbial community [58]. However, plant communities seemed to have minor impacts on bacterial diversity and it is in accordance with previous works [59,60]. Bacterial diversity was low with *P. pratensis* and *R. acetosella*, but no common feature was found between two plant communities in plant characteristics. Nonetheless, the unique OTUs present in specific communities suggest that the addition of plant species leads to the enhancement of bacterial biodiversity in the entire pasture. It also suggested that maintenance with various forage and weed communities could help promote plant-specific bacteria in pasture.

As there are no obvious trends in the effects of plant communities, bacterial diversity seemed to be more influenced by soil chemical properties. Soil pH is supposed to influence nutrient availability for microbes and it is positively correlated with bacterial diversity in many cases [18,61]. Diversity indices were more strongly correlated with available P than N nutrients, which is in accordance with a previous study [62]. The correlation analysis showed that NO_3_^−^ was more closely correlated with the major bacterial groups than NH_4_^+^. Zhang et al. [63] also obtained the same results in an investigation during succession on an abandoned farmland.

### 4.6. Putative Bacterial Function

The function of ecosystem engineers is critical for the productivity of pasture. However, diversity and community composition do not directly provide information about how bacteria contribute to ecological functions. Taxonomy and sequencing-based methods have been developed to link community data and ecological functions. FAPROTAX matched previous function data with taxonomic classifications of culturable bacteria [32]. FAPROTAX results showed that several functions were differently influenced by plant community and soil chemical properties. For example, cellulolysis differed with the plant communities but were not correlated with soil chemical properties. The production of cellulolytic enzyme is beneficial for rhizosphere colonisation and growth of cellulolytic bacteria can be promoted by plant cover [64]. Conversely, cellulolytic bacteria also have the potential to be plant growth-promoting rhizobacteria because cellulolysis transforms cellulose into sugars that are used as nutrients by various organisms [65]. These results implied that the plant community can alter soil process via functional bacteria. Meanwhile, nitrogen fixation was correlated with pH, and it indicates that the management of soil pH is important for soil fertility [66]. For ureolysis, many bacteria possess urease which degrades organic N and urea in agriculture, and it is influenced by nutrient levels [67]. These results suggest that ureolysis functions may decline with decreasing intensities of fertilisation.

PICRUSt is based on the sequencing of 16S rRNA, and pipelines have been developed to facilitate functional expectations [33]. However, performance of metagenome prediction for soil samples may be decreased [68]. Functions determined using KEGG pathways revealed that some material metabolisms were influenced both by plant communities and the soil properties. Degradation of toxic and xenobiotic pollutants including xylene and caprolactam reflect resistance to chemical disturbance in the soil ecosystem. Pollutant degradation is facilitated by the interactions between plants and rhizosphere microbes, which exemplify the role of plant community in this regard [69,70]. Some functions such as siderophore biosynthesis may promote plant growth by facilitating metal uptake [71]. Notably, carbon metabolism was correlated with pH and EC, suggesting that soil chemical properties are also important for the functional engineering of soil bacteria. Function expectations with KEGG pathways helped to assess the unculturable bacteria. More than 99% of soil bacteria are suggested to be unculturable, and 82.1% were unculturable in this study [72]. Hence, the analysis of putative functions using both FAPROTXA and PICRUSt systems is needed to fully address the changes in bacterial functions.

## 5. Conclusions

Difference in soil chemical properties due to topographic variation primarily altered both plant community and soil bacterial community in the pasture. Findings of bacterial groups that responded only at one specific plant community show that plants had individual effects on bacterial community, which is dissociated from soil effects. The greater number of uniquely present OTUs than absent OTUs suggests that a negative effect allows plants to affect specific bacteria. Soil chemical properties, including pH, EC and nutrients were the main factors driving bacterial putative functions. It is concerning that the decrease in soil nutrient levels due to decreased management may negatively influence some bacterial functions. Further studies are required to engineer functional bacterial groups for the practical management of pastures.

## Figures and Tables

**Table 1:** Plant and topographic characteristics for the five plant communities.

	Coverage (%)	Monocot /Dicot	Growth Form	Altitude (m)	Slope Degree (°)
Ag × Ph	80–98	monocot	tussock	793–832	16–33
Po	65–98	monocot	tussock	793–833	4–31
Fe	70–90	monocot	tussock	819–824	18–40
Ru	75–95	dicot	partial rosette	795–833	17–32
Sm × Ss	80–100	dicot	erect	795–817	42–55

Ag × Ph, *A. alba* × *P. pratense*; Po, *P. pratensis*; Fe, *F. ovina*; Ru, *R. acetosella*; Sm × Ss, *S. miyabei* × *S. salicifolia*.

**Table 2:** Chemical properties of soils.

	pH	EC (ds m^−1^)	C/N	NH_4_^+^-N (mg kg^−1^)	NO_3_^−^-N (mg kg^−1^)	Av. P (mg kg^−1^)
Ag × Ph	4.4 ± 0.1 ^ab^	0.5 ± 0.2 ^ab^	15.4 ± 1.7 ^a^	59.3 ± 10.4 ^a^	21.7 ± 12.0 ^a^	20.0 ± 2.1 ^ab^
Po	4.1 ± 0.2 ^b^	0.6 ± 0.1 ^a^	11.5 ± 0.5 ^c^	49.3 ± 13.6 ^a^	21.8 ± 6.9 ^a^	43.1 ± 3.2 ^a^
Fe	4.6 ± 0.1 ^a^	0.3 ± 0.0 ^b^	16.6 ± 0.6 ^a^	48.0 ± 16.0 ^a^	7.9 ± 0.5 ^b^	23.0 ± 10.2 ^ab^
Ru	4.2 ± 0.1 ^b^	0.5 ± 0.1 ^ab^	12.4 ± 0.1 ^bc^	54.5 ± 6.7 ^a^	12.6 ± 4.9 ^ab^	38.3 ± 5.2 ^a^
Sm × Ss	4.6 ± 0.1 ^a^	0.3 ± 0.1 ^b^	14.4 ± 2.0 ^ab^	61.6 ± 22.0 ^a^	10.3 ± 3.2 ^ab^	10.5 ± 3.4 ^b^

Data represent mean ± SD and different letters in the same column indicate significant differences according to Tukey test (*n* = 3, *p* < 0.05). Ag × Ph, *A. alba* × *P. pratense*; Po, *P. pratensis*; Fe, *F. ovina*; Ru, *R. acetosella*; Sm × Ss, *S. miyabei* × *S. salicifolia*.

**Table 3:** OTU numbers and alpha diversity of soil bacterial community.

		OTUs		Alpha Diversity Index	
	Species number (N)	Uniquely Present ^†^ (%) ^‡^	Uniquely Absent ^†^ (%) ^‡^	Shannon	Chao1
Ag × Ph	2000.1 ± 177.5 ^ab^	0.2	3.5	7.0 ± 0.1 ^a^	3915.3 ± 456.6 ^a^
Po	1714.0 ± 84.8 ^b^	0.3	8.5	6.5 ± 0.1 ^b^	2951.1 ± 303.1 ^b^
Fe	2098.7 ± 162.1 ^ab^	0.2	5.1	7.0 ± 0.2 ^a^	3855.5 ± 332.5 ^a^
Ru	1747.7 ± 148.4 ^b^	0.2	2.5	6.7 ± 0.1 ^ab^	3120.4 ± 244.8 ^b^
Sm × Ss	2196.0 ± 159.4 ^a^	0.5	6.2	7.0 ± 0.1 ^a^	3977.6 ± 239.6 ^a^

^†^ Uniquely present and absent OTUs indicate that the OTUs are found or missing for only one type of plant community, respectively. Data represent mean ± SD and different letters in the same column indicate significant differences according to Tukey test (*n* = 3, *p* < 0.05). ^‡^ Percentages were calculated using the data of major group (>0.01%). Ag × Ph, *A. alba* × *P. pratense*; Po, *P. pratensis*; Fe, *F. ovina*; Ru, *R. acetosella*; Sm × Ss, *S. miyabei* × *S. salicifolia*.

**Table 4:** Relative abundance of 14 conspicuous bacterial OTUs at the species level.

	Relative Abundance (%)				
	Ag × Ph	Po	Fe	Ru	Sm × Ss
*Afipia broomeae*	1.9 ± 0.3 ^a^	2.4 ± 0.1 ^a^	0.6 ± 0.4 ^b^	2.7 ± 0.9 ^a^	1.5 ± 0.1 ^ab^
FJ536874_s	0.8 ± 0.1 ^b^	5.8 ± 1.7 ^a^	0.0 ± 0.0 ^b^	1.7 ± 1.5 ^b^	0.0 ± 0.0 ^b^
AJ544784_s	1.7 ± 0.4 ^b^	4.5 ± 1.2 ^a^	0.2 ± 0.2 ^b^	1.8 ± 1.6 ^b^	0.1 ± 0.0 ^b^
AY913320_s	0.1 ± 0.1 ^b^	0.8 ± 0.1 ^a^	0.0 ± 0.0 ^b^	0.8 ± 0.4 ^a^	0.0 ± 0.0 ^b^
*Afipia felis*	0.3 ± 0.1 ^ab^	0.0 ± 0.0 ^c^	0.4 ± 0.1 ^a^	0.2 ± 0.1 ^bc^	0.3 ± 0.1 ^ab^
FJ466008_s	0.1 ± 0.1 ^bc^	0.5 ± 0.1 ^a^	0.1 ± 0.0 ^bc^	0.3 ± 0.1 ^b^	0.0 ± 0.0 ^c^
*Rhodanobacter soli*	1.0 ± 0.4 ^a^	0.1 ± 0.1 ^b^	0.0 ± 0.0 ^b^	0.1 ± 0.1 ^b^	0.0 ± 0.0 ^b^
DQ451441_s	0.1 ± 0.1 ^b^	0.1 ± 0.0 ^b^	0.6 ± 0.1 ^a^	0.1 ± 0.1 ^b^	0.2 ± 0.0 ^b^
4P002518_s	0.2 ± 0.1 ^b^	0.0 ± 0.0 ^b^	0.5 ± 0.1 ^a^	0.1 ± 0.1 ^b^	0.1 ± 0.1 ^b^
*Sporosarcina soli*	0.1 ± 0.1 ^b^	0.5 ± 0.1 ^a^	0.0 ± 0.0 ^b^	0.2 ± 0.1 ^b^	0.0 ± 0.0 ^b^
4P002219_s	0.0 ± 0.0 ^c^	0.5 ± 0.1 ^a^	0.0 ± 0.0 ^c^	0.2 ± 0.2 ^b^	0.0 ± 0.0 ^c^
*Parafrigoribacterium mesophilum*	0.4 ± 0.3 ^a^	0.0 ± 0.0 ^b^	0.0 ± 0.0 ^b^	0.0 ± 0.0 ^b^	0.0 ± 0.0 ^b^
EU150242_s	0.0 ± 0.0 ^b^	0.0 ± 0.0 ^b^	0.0 ± 0.0 ^b^	0.0 ± 0.0 ^b^	0.3 ± 0.1 ^a^
DQ058676_s	0.0 ± 0.0 ^b^	0.0 ± 0.0 ^b^	0.0 ± 0.0 ^b^	0.0 ± 0.0 ^b^	0.2 ± 0.1 ^a^

Data represent mean ± SD and different letters in the same row indicate significant differences according to Tukey test (*n* = 3, *p* < 0.05). Ag × Ph, *A. alba* × *P. pratense*; Po, *P. pratensis*; Fe, *F. ovina*; Ru, *R. acetosella*; Sm × Ss, *S. miyabei* × *S. salicifolia*.

## Data Availability

Not applicable.
